# Development of microsatellite markers for Hyacinth macaw (*Anodorhynchus hyacinthinus*) and their cross-amplification in other parrot species

**DOI:** 10.1186/s13104-015-1749-9

**Published:** 2015-12-01

**Authors:** Helder E. da Silva, Flavia T. Presti, Adriane P. Wasko, Danillo Pinhal

**Affiliations:** Department of Genetics, Institute of Biosciences, São Paulo State University (UNESP), Botucatu, São Paulo 18618-970 Brazil

**Keywords:** Microsatellites, Genetic diversity, Cross-species amplification, Conservation, Parrots

## Abstract

**Background:**

Hyacinth macaw *Anodorhynchus hyacinthinus* is the largest parrot of the world and is considered vulnerable to extinction due to its habitat loss and illegal trade associated to the international pet market demand. Genetic studies on this species are still incipient to generate a consistent characterization of the population dynamics and to develop appropriate conservation strategies. In this sense, microsatellite markers may support the detection of a population genetic structure for this bird species. However, at this time, none Hyacinth macaw species-specific primers for microsatellite loci have been so far established. This study aimed to develop and characterize polymorphic microsatellite markers for *A. hyacinthinus* and to check for their cross-amplification in other parrot species.

**Findings:**

Sequences containing repeated dinucleotide motifs were prospected and optimized from a genomic library that was enriched for microsatellites using magnetic beads. The analyses of 43–57 samples from wild individuals of three distinct Brazilian subpopulations led to the characterization of five polymorphic microsatellite loci. Allele richness per locus ranged from two to 12. Three loci exhibited observed heterozygosity values higher than 50 %, but the overall average value among all loci was close to 45 %. In addition, successful primer cross-amplification was verified in seven other investigated species of Neotropical parrots.

**Conclusions:**

The newly developed markers have shown to be potentially useful for in situ and ex situ population studies to support future conservation actions of Hyacinth macaw and other parrots.

## Findings

Hyacinth macaw *Anodorhynchus hyacinthinus* (Latham, 1790; Psittaciformes, Psittacidae) is the largest parrot of the world and has great importance in conservation biology due to its flagship species status [1, 2]. In Brazil, the species is mainly distributed in three non-continuous regions that correspond to different biomes, exhibiting dissimilar faunal and floral compositions. In these areas, Hyacinth macaw has different preference for food items and nesting sites [3, 4]. Moreover, their populations are sensitive to the continuous habitat loss and have also been directly affected by the illegal trade, which led this species to be considered as vulnerable to extinction [5]. Genetic studies have gained prominence to aid in the conservation of a wide-range endangered vertebrates [6–9], including bird species [6]. Among the genetic markers, microsatellite loci have shown to be useful in variable contexts, notably to estimate the influence of genetic components in population studies [10], to trace the geographic origin of unknown individuals [11] or to determine the kinship of siblings [12], among other applications. Currently, there is a very limited source of microsatellite loci characterized in Neotropical parrots, despite of the species richness of this group [13]. Available studies on Hyacinth macaw have used nonspecific microsatellite markers to assess the genetic variability of this species [14], their population genetic structure [15, 16] and to identify the probable geographic origin of a rescued individual from captivity [16]. Despite one pair of primers that was developed for *A. hyacinthinus* (Davis S, unpublished data), all these studies used heterologous primers characterized for other parrot species such as *Ara ararauna* [17, 18], *Amazona guildingii* [19, 20], and *Psittacus erithacus* [21]. However, these other birds do not represent sister taxa of the genus *Anodorhynchus* and are not closely interrelated [22, 23]. In this sense, the use of heterologous microsatellite primers generally leads to a decrease in the polymorphism level as the phylogenetic distance among species increases [24–26]. In fact, for Hyacinth macaw, the number of alleles and observed heterozygosity for all analysed heterologous *loci* were considerably lower when compared to those species from which the primers were originally described [16]. In addition, a lower genetic variability has been observed in threatened parrots when compared to other non-threatened species [14]. So far, there are no species-specific primers for microsatellite loci available for Hyacinth macaw described in literature. Thus, considering the vulnerable status of this species, a proper assessment of the genetic background of extant populations requires the analysis of a considerable number of *loci*. Consequently, the development of microsatellite specific primers for *A. hyacinthinus* could enlarge the rol of available molecular tools for the accurate assessment of its genetic diversity and population structure. Thereby, in this study we report the isolation and characterization of microsatellite loci for Hyacinth macaw and their cross-amplification in other species of Neotropical parrots.

Total genomic DNA from 57 individuals of *A. hyacinthinus* from distinct Brazilian regions (Table 1) was isolated following the extraction protocol using proteinase K and phenol–chloroform [27]. A DNA sample of one animal was used on a digestion assay and fragments between 700 and 1200 bp were selected by digestion with the restriction enzyme *Afa*I (Invitrogen) and further ligated to adapters with known sequences (Rsa21 and Rsa25). Then these fragments were enriched throughout hybridization to probes containing dinucleotide repeats (GT and CT) chemically linked to biotin and selected using magnetic beads covered with streptavidin. Subsequently, the recovered fragments were inserted into a cloning vector pGEM-T Easy (Promega), and electroporation was used to transform competent cells of *Escherichia coli* (XL1-Blue). Then, plasmid extraction was performed using the alkaline lysis method [28], and T7 and SP6 primers were used for sequencing the library inserts with an ABIPrism 3500xL Genetic Analyzer (Applied Biosystems). The protocol used for microsatellite enrichment technique was described in Billotte et al. [29] with some modifications. Generated nucleotide sequences were screened for microsatellites regions in the program SSRIT [30]. Primer3 plus [31] and OligoAnalyzer 3.1 (Integrated DNA Technologies) softwares were used for the design of the species-specific primers and to verify the occurrence of hairpins, self-dimers, and heterodimers. PCR conditions were as follows: initial denaturation at 95 °C for 10 min, followed by 35 cycles at 95 °C for 1 min, 45–65 °C for 40 s (nine annealing temperatures were applied: 45, 53, 55, 57, 59, 60, 61, 63, and 65 °C), 72 °C for 40 s, and a final extension at 72 °C for 10 min. Each PCR contained 20–50 ng of DNA, 0.1 μL of *Taq*DNA polymerase (5 U/μL, Invitrogen), 1 μL of each primer (10 mM), 0.4 μL of MgCl_2_ (25 mM), 1.2 μL of PCR buffer (10×), 1 μL of dNTPs (2.5 mM), and autoclaved Milli-Q water to complete 12 μL. Approximately 3 μL of the products were electrophoresed through 1.5 % agarose gels to verify amplification success.

**Table 1:** Sample data used for the microsatellite loci characterization in Hyacinth macaw

Subpopulation	Location	NT	NL	Field collection year
Pará (PA)	Canaã dos Carajás	2	1–2	2013
		1	1	2009
		9	5–9	2008
		5	0–3	2007
	Parque Zoobotânico (Parauapebas)	6	6	2013
		1	0–1	2007
	Rio Itacaiúnas (FLONA Itacaiúnas)	1	1	2013
	Fundação Zoobotânica (Marabá)	1	0–1	2007
	Redenção	1	1	1997
	Parque Zoobotânico (Belém)	2	0–2	–
	Rio Iriri (Altamira)	1	0–1	–
	Serra dos Carajás (Serra Norte)	1	0–1	–
	Capitão Poço	1	0–1	–
	–	1	0–1	–
		33	20–27	
Northest (NE)	Piauí^a^	4	1–4	1999
	Tocantins^a^	11	3–10	–
		15	5–14	
Pantanal (PN)	Rio Negro, MS	1	0–1	2001–2002
	Nhecolândia, MS	3	1–3	2000–2002
	Abobral, MS	3	2–3	2000
	Barão do Melgaço, MT	5	2–5	2009
		5	4–5	2002–2004
		17	7–17	
Unknown	Fundação Lymington	3	0–3	2013
Total		68	43–57	

*NT* total number of samples used considering all *loci* separately, *NL* variation in the number of samples used considering each locus independently

^a^Unknown exact locality

–: Unknown data

For the genetic analyses, we collected approximately 0.1 mL of peripheral blood from the brachial veins of the lower wing of chicks found in natural nests, using disposable syringes and needles. The nests were accessed by an alpinism technique adapted to trees. Subsequently, the blood samples were transferred into microtubes containing approximately 0.5 mL of absolute ethanol and kept at air temperature. In addition, several research partners provided samples that were collected in previous field campaigns (details in Table 1). This work was approved by the federal government authoritative (IBAMA; permission Number: 36590-1). Microsatellite markers that yielded a clear amplification band on agarose gels were then tested for polymorphism level using 43–57 samples of *A. hyacinthinus* belonging to three potentially sub-populations of this species (Table 1). The polymorphism level was determined by a genotyping method throughout PCR amplification of the DNA samples with a sequence-specific forward primer composed by a M13 (−21) tail with distinct fluorescent dye labels (HEX or FAM, Applied Biosystems) [32], following a previously established protocol [33]. Fragments genotyping was performed in ABIPrism 3500xL or ABIPrism 3130xL Genetic Analyzers (Applied Biosystems) in order to detect the alleles size. Each reaction contained 0.1 μL of *Taq*DNA polymerase (5 U/μL, Invitrogen), 0.1 μL of the forward primer with a M13 tail (5′CACGACGTTGTAAAACGAC-3′) (10 μM) [33], 0.3 μL of the reverse primer (10 μM), 0.2 μL of fluorescent dye label (10 mM), 0.4 μL of MgCl_2_ (25 mM), 1 μL of dNTPs (4 mM), 1.2 μL of PCR buffer (10×), 20–50 ng of DNA, and autoclaved Milli-Q water to complete 12 μL. PCR conditions were as follows: initial denaturation at 95 °C for 10 min, followed by 35 cycles at 95 °C for 1 min, 57–60 °C for 40 s (as detailed in Table 2), and 72 °C for 40 s, followed by 10 M13 tail cycles at 95 °C for 1 min, 55 °C for 40 s, 72 °C for 40 s, and a final extension step at 72 °C for 10 min. Approximately 2 μL of the products were electrophoresed through 1.5 % agarose gels to evaluate amplification success and to estimate their concentration. An aliquot of 1.0 μL of each amplified product was mixed with 0.2 μL of a molecular marker GeneScan ™ ROX ™-500 STANDARD (2 fmol, Applied Biosystem) and Hi-Di™ formamide (Applied Biosystems) to complete 10 μL of reaction. The mixture was analyzed in automatic sequencer ABIPrism 3500 (Applied Biosystems). The peaks and the sizes of the fragments for each allele were obtained by GeneMarker v2.6.3. The Genepop 4.2 program [34] was used to check the number of alleles per locus, linkage disequilibrium between loci, expected heterozygosity (He), observed heterozygosity (Ho), inbreeding coefficient (Fis) and Hardy–Weinberg equilibrium. The software Cervus was used to estimate the PIC (polymorphic information content) of each locus and the Micro-checker program [35] was used to assess the presence of null alleles, genotyping errors, and allele dropout. Additionally, we tested the transferability of the described primer sets in other seven Neotropical parrot species (*Amazona guildingii*, *A. ochrocephala*, *Ara severus*, *A. macao*, *A. ararauna*, *A. chloropterus*, and *Anodorhynchus leari*). For this purpose, PCR conditions were as follows: an initial denaturation step at 95 °C for 10 min, followed by 10 cycles at 95 °C for 1 min, 60 °C touchdown decreasing 0.5 °C at every cycle, and 72 °C for 40 s, followed by 25 cycles at 95 °C for 1 min, 55 °C for 40 s, 72 °C for 40 s and a final extension at 72 °C for 10 min. Each PCR contained 20–50 ng of DNA, 0.2 μL of *Taq*DNA polymerase (5 U/μL, Invitrogen), 1 μL of each primer (10 mM), 0.4 μL of MgCl_2_ (25 mM), 1.2 μL of PCR buffer (10×), 1 μL of dNTPs (2.5 mM), and autoclaved Milli-Q water to complete 12 μL. An aliquot of 3 μL of each product was electrophoresed in 1.5 % agarose gels to evaluate amplification success.

**Table 2:** Characterization of species-specific primers developed for Hyacinth macaw

Locus	Primers sequences (5′–3′)	Repeat motif	Size range	T °C	N	n	Ho	He	Fis	PIC
AnH6	F AAAGGCAGTTCAGGTGTTGG R ACACACACGCACATACTCCA	(GT)n(AT)n(GT)n	234–236	60–55 °C (td)	44	2	0.500	0.502	0.003	0.373
AnH10	F CCTATACCCAGCTCCCAACA R AGCCTTCAGTGGCTCATTGT	(AC)×9	166–172	57 °C	57	3	0.175	0.193	0.092	0.179
AnH17	F TTCCCATTGGATATCTTGTCAG R ATTGGCAATGGCCTAAACAC	(CA)×16	184–192	59 °C	49	5	0.531	0.669	0.217	0.605
AnH23	F TGTGGCATCTGTAAAGAAAGAGG R GCCTGGGGAGTGATTGTTTA	(AC)×9	222	57 °C	18	1^a^	–	–	–	–
AnH33	F GCCTGTGCCAGATGGTAAAT R GCCCTAAAAATGCTTTCCAA	(TG)×12	177–179	60–55 °C (td)	51	2	0.275	0.363	0.237	0.295
AnH34	F GACAGACACATCCGCTTCAA R AACACACATCTTCATATGCAACC	(TG)×24	172–210	60 °C	43	12	0.721	0.818	0.119	0.786
				Mean		4.8	0.440	0.509	0.134	0.448

Microsatellite loci, sequence of primers, repeat motifs, fragment size, annealing temperature (T °C), number of used samples (N), number of alleles (n), observed heterozygosity (Ho), expected heterozygosity (He), inbreeding coefficient (Fis) and PIC (polymorphic information content)

*td* touchdown decreasing 0.5 °C per cycle

^a^Monomorphic loci

From a library composed by a total of 96 bacterial colonies, 52 clones (54.17 %) were recovered and sequenced. The majority of the generated sequences, however, were not suitable for primers design or possessed a low number of repeat motifs and therefore had to be discarded. From this filtering step, only six clones could be used for the primers design (11.53 %).

As outcome, with the exception of the AnH23 locus that was found to be monomorphic, five isolated microsatellite loci were polymorphic in *A. hyacinthinus* (Table 2). The number of alleles per locus varied from two (AnH6 and AnH33) to 12 (AnH34), the observed heterozygosity ranged from 0.175 (AnH10) to 0.721 (AnH34), with an average of 0.440 for all loci, and the expected heterozygosity per locus ranged from 0.193 (AnH10) to 0.818 (AnH34) with an average of 0.509 between all loci (Table 2). All analyzed polymorphic loci have shown no evidence of linkage disequilibrium (p > 0.05) and no locus was found in Hardy–Weinberg disequilibrium (p > 0.01). In the analysis carried out in the Micro-checker program [35], the presence of null alleles was not detected. In addition, among the five polymorphic loci, two (AnH6 and AnH33) were considered reasonably informative (PIC between 0.25 and 0.5) and two (AnH17 and AnH34) were highly informative (PIC > 0.5) (Table 2) [36]. Furthermore, the designed primers for Hyacinth macaw generated amplification products for other seven species of Neotropical parrots. The primer sets AnH6, AnH33, and AnH17 led to amplification results for two, three, and five species, respectively, whereas both AnH10 and AnH34 primer sets were effective for six species (Table 3). Although the AnH23 locus was monomorphic in *A. hyachintinus*, primers for this microsatellite also generated amplification products for all the other tested parrots and their polymorphism should be further investigated. Curiously, primers for the AnH6 locus did not lead to amplification results in *A. leari* that is the phylogenetically closest species to Hyacinth macaw, probably due to specific mutations in the primers annealing regions. The described primers in this work have proven to be functional and can serve as important tools to determine the variability and the genetic structure of populations of Hyacinth macaw and other Neotropical parrots and may assist in in situ and ex situ conservation plans.

**Table 3:** Cross amplification of *Anadorhynchys hyacinthinus* microsatellite loci in other seven species of Neotropical parrots

Species	Locus					
	AnH6	AnH10	AnH17	AnH23	AnH33	AnH34
*Amazona guildingii*	−	+	−	+	−	+
*Amazona ochrocephala*	−	−	−	+	−	−
*Ara severus*	−	+	+	+	+	+
*Ara macao*	−	+	+	+	−	+
*Ara ararauna*	+	+	+	+	−	+
*Ara chloropterus*	+	+	+	+	+	+
*Anodorhynchus leari*	−	+	+	+	+	+

+ Amplified successfully

− Not amplified

## Availability of supporting data

The microsatellites data supporting the results of this article are available in GenBank at NCBI (http://www.ncbi.nlm.nih.gov/genbank/). Accession Numbers KP860340 to KP860345.
